# Inhibition of ALDH2 by *O*-GlcNAcylation contributes to the hyperglycemic exacerbation of myocardial ischemia/reperfusion injury

**DOI:** 10.18632/oncotarget.14297

**Published:** 2016-12-27

**Authors:** Baoshan Liu, Jiali Wang, Minghua Li, Qiuhuan Yuan, Mengyang Xue, Feng Xu, Yuguo Chen

**Affiliations:** ^1^ Department of Emergency, Qilu Hospital, Shandong University, Jinan, China; ^2^ Chest Pain Center, Qilu Hospital, Shandong University, Jinan, China; ^3^ Key Laboratory of Emergency and Critical Care Medicine of Shandong Province, Qilu Hospital, Shandong University, Jinan, China; ^4^ Key Laboratory of Cardiovascular Remodeling & Function Research, Chinese Ministry of Education & Chinese Ministry of Public Health, Qilu Hospital, Shandong University, Jinan, China

**Keywords:** myocardial ischemia/reperfusion, hyperglycemia, ALDH2, *O*-GlcNAcylation

## Abstract

Although hyperglycemia is causally related to adverse outcomes after myocardial ischemia/reperfusion (I/R), the underlying mechanisms are largely unknown. Here, we investigated whether excessive *O*-linked-*N*-acetylglucosamine (*O*-GlcNAc) modification of acetaldehyde dehydrogenase 2 (ALDH2), an important cardioprotective enzyme, was a mechanism for the hyperglycemic exacerbation of myocardial I/R injury. Both acute hyperglycemia (AHG) and diabetes (DM)-induced chronic hyperglycemia increased cardiac dysfunction, infarct size and apoptosis index compared with normal saline (NS)+I/R rats (*P*<0.05). ALDH2 *O*-GlcNAc modification was increased whereas its activity was decreased in AHG+I/R and DM+I/R rats. High glucose (HG, 30mmol/L) markedly increased ALDH2 *O*-GlcNAc modification compared with Con group (5mmol/L) (*P*<0.05). ALDH2 *O*-GlcNAc modification was increased by 62.9% in Con+PUGNAc group whereas it was decreased by 44.1% in Con+DON group compared with Con group (*P*<0.05). Accordingly, ALDH2 activity was decreased by 18.1% in Con+PUGNAc group whereas it was increased by 17.9% in Con+DON group. Moreover, DON decreased levels of 4-hydroxy-2-nonenal (4-HNE), aldehydes, protein carbonyl accumulation and apoptosis index compared with HG+H/R group (*P*<0.05). Alda-1, a specific activator of ALDH2, significantly decreased ALDH2 *O*-GlcNAc modification and improved infarct size, apoptosis index and cardiac dysfunction induced by I/R combined with hyperglycemia. These findings demonstrate that ALDH2 *O*-GlcNAc modification is a key mechanism for the hyperglycemic exacerbation of myocardial I/R injury and Alda-1 has therapeutic potential for inducing cardioprotection.

## INTRODUCTION

Acute myocardial infarction (AMI) is the leading cause of morbidity and mortality worldwide [1]. The prognosis of patients with AMI is conditioned by a series of risk factors which rend the process more complex. Among them, hyperglycemia in AMI patients with or without previous diabetes worsens the prognosis [2]. Strong evidence demonstrates that elevation of plasma glucose independently exacerbates cardiac dysfunction, infarct size and cardiomyocyte apoptosis following myocardial ischemia/reperfusion (I/R) [3–6]. Despite these observations, the underlying mechanisms by which hyperglycemia worsens myocardial I/R injury are incompletely understood.

Excessive elevation of plasma glucose could cause protein *O*-linked-*N*-acetylglucosamine (*O*-GlcNAc) modification through the hexosamine biosynthesis pathway which is a common post-translational modification that occurs on serine/threonine residues of proteins [7, 8]. The *O*-GlcNAcylation of specific proteins could modulate their structure and function and thus influence signaling cascades, antioxidant defenses, *et al* [9–12]. Up to now, there are numerous reports on *O*-GlcNAcylation of cytosolic, nuclear and mitochondrial proteins distributed in various tissues [13–15]. However, whether *O*-GlcNAc modification of specific responsible proteins contributes to hyperglycemic exacerbation of myocardial I/R injury has not been investigated.

Mitochondrial aldehyde dehydrogenase2 (ALDH2) has recently been identified as an important cardioprotective enzyme, whose activity inversely correlates with infarct size. Enhancement of ALDH2 activity reduces infarct size induced by I/R, whereas inhibition of ALDH2 activity worsens infarct size [16, 17]. ALDH2-mediated detoxification of cytotoxic aldehydes, including 4-hydroxy-2-nonenal (4-HNE), represents a possible cytoprotective mechanism that enhances tissue survival in the heart during I/R. Our and other studies have demonstrated that ALDH2 could be inactivated by hyperglycemia [18, 19]. However, whether *O*-GlcNAcylation of ALDH2 could be a mechanism for its activity down-regulation and resultant aggravation of myocardial I/R injury under hyperglycemia conditions has not been elucidated, despite candidates serine/threonine residues of ALDH2 protein exist [20, 21].

Here, the aims of the present study are to (1) investigate whether ALDH2 is *O*-GlcNAcylated excessively under hyperglycemic conditions, and (2) determine the role of ALDH2 *O*-GlcNAcylation in the hyperglycemic exacerbation of myocardial I/R injury.

## RESULTS

### Hyperglycemia exacerbated myocardial I/R-induced injury in rats

There was no significant differences in plasma glucose levels among Sham, NS+I/R and acute hyperglycemia (AHG) +I/R groups (plasma glucose conc: 5.3±1.5mmol/L, 5.1±2.5mmol/L and 6.2±2.5mmol/L, respectively), whereas the mean plasma glucose concentration was 15.6±3.4mmol/L in diabetic rats at baseline condition. For AHG+I/R group rats, plasma glucose increased to more than 20mmol/L instantly after the bolus injection of glucose, and maintained over 20mmol/L during myocardial I/R procedure.

**Figure 1:**
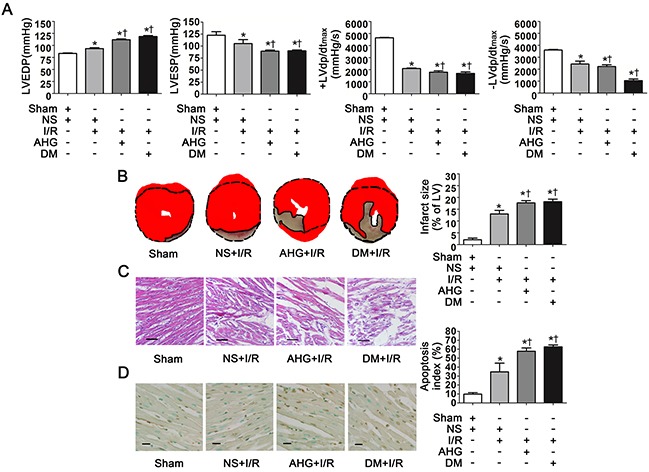
Hyperglycemia exacerbated cardiac dysfunction, increased infarct size and apoptosis index after myocardial ischemia/reperfusion in rats **A**. LVEDP, LVESP, +LVdp/dt_max_ and -LVdp/dt_max_. LVEDP, left ventricular end diastolic pressure; LVESP, left ventricular end systolic pressure; +LVdp/dt_max_, left ventricular maximal rate of pressure increase; -LVdp/dt_max_, left ventricular maximal rate of pressure decrease. **B**. Left: representative photographs of heart sections stained by 2, 3, 5-triphenyltetrazolium (TTC); Right: myocardial infarct size expressed as percentage of the total left ventricular area. **C**. Representative photographs of myocardial tissue with H&E staining. Scale bar, 200μm. **D**. Left: representative photographs of in situ detection of apoptotic myocytes by TUNEL staining; Right: percentage of TUNEL-positive nuclei in heart tissue sections. Scale bar, 50μm. Sham, sham-operated; NS, normal saline; I/R, myocardial ischemia/reperfusion; AHG, acute hyperglycemia; DM, diabetes. Data are means ± SEM of 8 to 10 samples in each group. *P<0.05 vs Sham group; †*P*<0.05 vs NS+I/R group.

Thirty minutes of ischemia followed by 90min of reperfusion resulted in significant increase in left ventricular end diastolic pressure (LVEDP) and decrease in left ventricular end systolic pressure (LVESP), left ventricular maximal rate of pressure increase (+LVdp/dt_max_) and left ventricular maximal rate of pressure decrease (−LVdp/dt_max_) compared with Sham controls (*P*<0.05, respectively. Figure 1A). Both AHG and diabetes (DM)-induced chronic hyperglycemia further significantly increased LVEDP and decreased LVESP, +LVdp/dt_max_ and –LVdp/dt_max_ compared with NS+I/R rats (*P*<0.05, respectively. Figure 1A).

As expected, there was an obvious myocardial infarct size area in NS+I/R rats whereas it was nearly null in Sham controls (Figure 1B). AHG and DM prominently exacerbated infarct size area compared with NS+I/R rats (*P*<0.05, respectively. Figure 1B). HE staining showed that myocardial injury at the tissue level in the NS+I/R rats was markedly greater than Sham controls. The injury was further increased in both AHG+I/R and DM+I/R groups (Figure 1C).

Finally, we determined myocardial apoptosis since it plays a critical role in cardiomyocyte loss and cardiac dysfunction after I/R [22–24]. The results showed that the apoptosis index was obviously increased in NS+I/R rats compared with Sham controls (*P*<0.05, Figure 1D). AHG and DM-induced chronic hyperglycemia further increased apoptosis index (*P*<0.05, respectively. Figure 1D).

Taken together, our results confirmed that hyperglycemia could exacerbate myocardial I/R injury including cardiac dysfunction and increased infarct size and apoptosis, consistent with previous studies [3, 5]. Interestingly, both AHG and DM-induced chronic hyperglycemia significantly increased myocardial I/R injury to the same extent.

### ALDH2 *O*-GlcNAc modification was increased after myocardial I/R in rats with hyperglycemia

**Figure 2:**
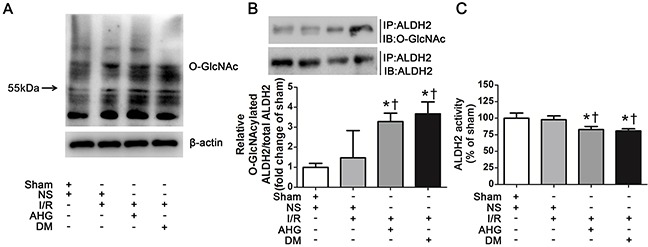
Hyperglycemia increased myocardial ALDH2 *O*-GlcNAc modification and decreased its activity after myocardial ischemia/reperfusion in rats **A**. Overall protein *O*-GlcNAcylation in myocardial tissue. Tissue lysates were separated by SDS-PAGE, and western blot was performed using specific anti-*O*-GlcNAc (CDT110.6) antibody. **B**. ALDH2 *O*-GlcNAcylation in myocardial tissue. Tissue lysates were immunoprecipitated with specific anti-ALDH2 antibody and then subjected to western blot using anti-*O*-GlcNAc (CDT110.6) antibody. **C**. ALDH2 dehydrogenase activity analysis. ALDH2 activity was determined by measuring the conversion of NAD^+^ to NADH. In each graph, the mean value of Con was expressed as 1 or 100%. IP, immunoprecipitation; IB, immunoblotting; Sham, sham-operated; NS, normal saline; I/R, myocardial ischemia/reperfusion; AHG, acute hyperglycemia; DM, diabetes. Data are means ± SEM of 4 to 5 samples in each group. *P<0.05 vs Sham group; †*P*<0.05 vs NS+I/R group.

To examine whether ALDH2 could be *O*-GlcNAc modified under high glucose condition during myocardial I/R procedure, we first measured total protein *O*-GlcNAc modification level in wild-type rat hearts. Various proteins showed marked *O*-GlcNAc modification, indicating *O*-GlcNAc modification is a common post-translational modification. Myocardial I/R per se did not increase overall *O*-GlcNAc modification of proteins. However, the total protein *O*-GlcNAcylation was apparently increased in AHG+I/R and DM+I/R groups, compared with Sham and NS+I/R groups, respectively (Figure 2A).

Next, we measured ALDH2 *O*-GlcNAc modification through immunoprecipitation and western blot analysis. The results showed that ALDH2 *O*-GlcNAc modification was apparent in Sham control rats. It was slightly increased in NS+I/R group compared with Sham controls, but the difference did not reach significant level. However, ALDH2 *O*-GlcNAc modification was significantly increased by 2.29 fold in AHG+I/R group and it was enhanced by 2.67 fold in DM+I/R group compared with NS+I/R group (*P*<0.05, respectively. Figure 2B). There was no significant difference in ALDH2 *O*-GlcNAc modification between AHG+I/R rats and DM+I/R rats.

We measured *O*-GlcNAc modification of other mitochondrial proteins including COX I and NDUFA9 as positive controls whose *O*-GlcNAc modification was confirmed in previous report [14]. Our results showed that the COX I and NDUFA9 *O*-GlcNAc modification was similar in Sham controls and NS+I/R rats, whereas it was significantly increased in DM+I/R group (*P*<0.05, Supplementary Figure 1). These results proved the reliability of our results.

Protein *O*-GlcNAc modification may alter its function [10]. After observing ALDH2 *O*-GlcNAc modification, we further measured its function through evaluating the activity. We found that ALDH2 activity was similar between Sham and NS+I/R groups, whereas it was decreased by 17.2% in AHG+I/R rats and by 19.2% in DM+I/R rats in comparison with NS+I/R rats (*P*<0.05, respectively. Figure 2C). As previous reports showed that protein *O*-GlcNAc modification could regulate gene expression, we detected the level of ALDH2 expression and found that it changed in neither AHG+I/R nor DM+I/R conditions compared with NS+I/R group (Supplementary Figure 2). Thus, these data proved that *O*-GlcNAc modification of ALDH2 could be enhanced after myocardial I/R in rats with hyperglycemia, which was inversely related with its activity.

### Hyperglycemia increased ALDH2 *O*-GlcNAc modification

**Figure 3:**
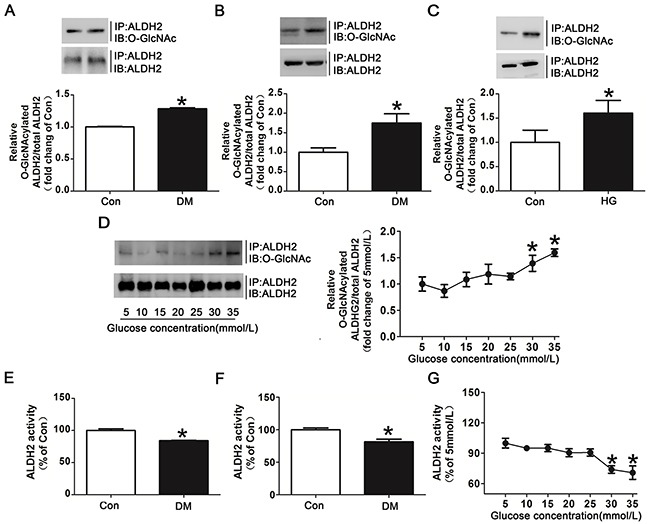
Hyperglycemia increased ALDH2 *O*-GlcNAc modification and decreased its activity both *in vivo* and *in vitro* **A**. ALDH2 *O*-GlcNAcylation in myocardial tissue of diabetic patients and non-diabetic controls. Tissue lysates were immunoprecipitated with specific anti-ALDH2 antibody and then subjected to western blot using anti-*O*-GlcNAc (CDT110.6) antibody. **B**. ALDH2 *O*-GlcNAcylation in myocardial tissues of diabetic rats and non-diabetic controls. **C**. ALDH2 *O*-GlcNAcylation in H9c2 cells treated with high glucose (HG, 30mmol/L) or normal glucose (Con, 5mmol/L) for 48h. **D**. Dose-dependent effect of glucose on ALDH2 *O*-GlcNAcylation. The glucose concentrations ranged from 5mmol/L to 35mmol/L. **E**. ALDH2 dehydrogenase activity in myocardial tissues of diabetic patients and non-diabetic controls. ALDH2 activity was determined by measuring the conversion of NAD^+^ to NADH. **F**. ALDH2 dehydrogenase activity in myocardial tissues of diabetic rats and non-diabetic controls. **G**. Dose-dependent effect of glucose on ALDH2 activity. The glucose concentration ranged from 5mmol/L to 35mmol/L. Mannitol was used to keep the osmolarity consistent among different glucose concentrations *in vitro*. In each graph, the mean value of Con was expressed as 1 or 100%. IP, immunoprecipitation; IB, immunoblotting. Data are means ± SEM of 4 to 5 samples in each group. *P<0.05 vs Con group or 5mmol/L group.

Since myocardial I/R per se could not increase ALDH2 *O*-GlcNAc modification significantly (Figure 2B), we further examined whether hyperglycemia could increase myocardial ALDH2 *O*-GlcNAc modification independently. Human studies showed that ALDH2 *O*-GlcNAc modification was moderately increased by 28.3% in the diabetic patients compared with non-diabetic controls (*P*<0.05, Figure 3A). A similar tendency was observed with the comparison between the diabetic rats and wild-type rats (*P*<0.05, Figure 3B).

In addition to human and animal studies, we also determined the effect of high glucose on ALDH2 *O*-GlcNAc modification *in vitro*. The results showed that high glucose treated for 24h did not significantly increase ALDH2 *O*-GlcNAc modification compared with normal controls (data not shown). However, high glucose treated for 48h markedly increased ALDH2 *O*-GlcNAc modification compared with normal controls (with 60.2% increase for 48h, *P*<0.05, Figure 3C). Similarly, *O*-GlcNAcylated COX I and NDUFA9 were also increased in high glucose treatment for 48h (Supplementary Figure 3). ALDH2 *O*-GlcNAc modification was increased gradually with significance occurred at 30mmol/L and 35mmol/L as the concentrations of glucose were increased from 5mmol/L to 35mmol/L (Figure 3D). These data suggested that hyperglycemia increased ALDH2 *O*-GlcNAc modification in a time-and dose-dependent manner.

Moreover, ALDH2 activity was decreased by 15.9% and 19.6% in diabetic patients and animals compared with their controls (*P*<0.05, respectively. Figure 3E, 3F). The decreased ALDH2 activity was also observed as the concentrations of glucose were increased. The significant decrease of ALDH2 activity occurred at the glucose concentration of 30mmol/L and 35mmol/L (*P*<0.05, Figure 3G), suggesting an inverse correlation between ALDH2 *O*-GlcNAc modification and its activity.

### ALDH2 *O*-GlcNAc modification modulated its activity

**Figure 4:**
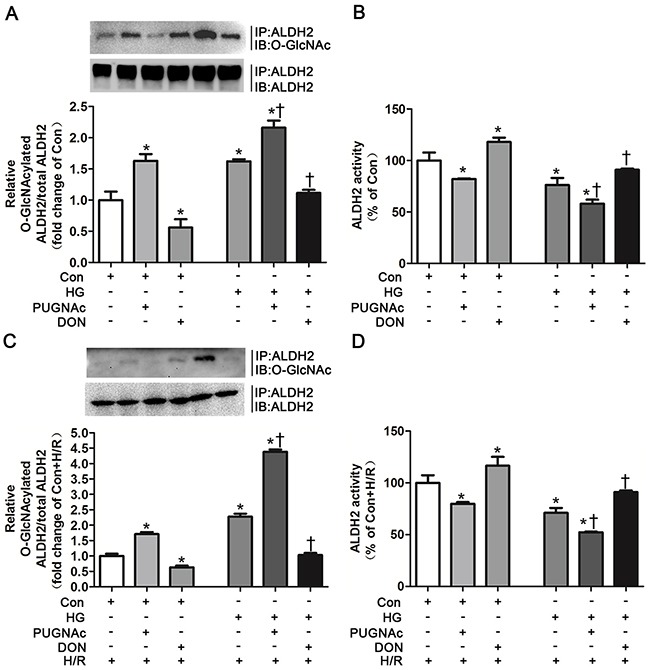
*O*-GlcNAc modification of ALDH2 resulted in its activity decrease **A**. ALDH2 *O*-GlcNAcylation in Con, Con+PUGNAc and Con+DON, and HG, HG+PUGNAc and HG+DON groups *in vitro*. Cell lysates were immunoprecipitated with specific anti-ALDH2 antibody and then subjected to western blot using anti-*O*-GlcNAc (CDT110.6) antibody. **B**. ALDH2 dehydrogenase activity in Con, Con+PUGNAc and Con+DON, and HG, HG+PUGNAc and HG+DON groups *in vitro*. ALDH2 activity was determined by measuring the conversion of NAD^+^ to NADH. **C**. ALDH2 *O*-GlcNAcylation in Con+H/R, Con+PUGNAc+H/R and Con+DON+H/R, and HG+H/R, HG+PUGNAc+H/R and HG+DON+H/R groups *in vitro*. **D**. ALDH2 dehydrogenase activity in Con+H/R, Con+PUGNAc+H/R and Con+DON+H/R, and HG+H/R, HG+PUGNAc+H/R and HG+DON+H/R groups *in vitro*. Mannitol was used to keep the osmolarity consistent among different glucose concentrations *in vitro*. In each graph, the mean value of Con or Con+H/R was expressed as 1 or 100%. IP, immunoprecipitation; IB, immunoblotting; Con: normal glucose; HG: high glucose; PUGNAc: a specific *O*-GlcNAc modification enhancer; DON: a specific *O*-GlcNAc modification inhibitor; H/R: hypoxia/reoxygenation. Data are means ± SEM of 4 to 5 samples in each group. *P<0.05 vs Con group, †*P*<0.05 vs HG group in normal oxygen conditions or H/R conditions.

We investigated whether ALDH2 *O*-GlcNAc modification could directly modulate its activity using specific *O*-GlcNAc modification enhancer PUGNAc and inhibitor DON *in vitro*, respectively. PUGNAc treated for 48h significantly increased ALDH2 *O*-GlcNAc modification by 62.9% compared with basal conditions (control group), whereas DON markedly decreased ALDH2 *O*-GlcNAc modification by 44.1% (*P*<0.05, Figure 4A). Accordingly, ALDH2 activity was decreased by 18.1% in the presence of PUGNAc in comparison with basal conditions, whereas its activity was increased by 17.9% in the presence of DON (*P*<0.05, Figure 4B).

ALDH2 *O*-GlcNAc modification was increased by 1.16 fold in the HG+PUGNAc group compared with basal conditions, whereas hyperglycemia per se increased ALDH2 *O*-GlcNAc modification by 66.2%, indicating that PUGNAc could further increase ALDH2 *O*-GlcNAc modification under high glucose conditions (Figure 4A). ALDH2 *O*-GlcNAc modification was decreased to the levels that similar to basal conditions in the HG+DON group (Figure 4A). Moreover, ALDH2 activity was significantly decreased in the presence of high glucose compared with basal conditions (*P*<0.05). HG+PUGNAc group further decreased ALDH2 activity, whereas ALDH2 activity in HG+DON group was enhanced to the levels in basal conditions (*P*<0.05, respectively. Figure 4B).

We further confirmed whether PUGNAc and DON could modulate ALDH2 activity in the H/R conditions. H9c2 cells were randomly treated in basal (Con), Con+PUGNAc, Con+DON, high glucose (HG), HG+PUGNAc and HG+DON conditions. Cells in all groups were treated with H/R. Similar tendency of alteration for ALDH2 *O*-GlcNAc modification among the groups was observed, but the percentage of alteration was greater among the groups under H/R conditions compared with those under normal oxygen conditions. PUGNAc increased ALDH2 *O*-GlcNAc modification by 70.1% and DON decreased ALDH2 *O*-GlcNAc modification by 36.7% in comparison with basal condition when treated under H/R (*P*<0.05, respectively. Figure 4C). HG+PUGNAc increased ALDH2 *O*-GlcNAc modification by 3.38 fold and HG+DON decreased ALDH2 *O*-GlcNAc modification by 1.25 fold in comparison with HG condition when treated under H/R (*P*<0.05, respectively. Figure 4C). Similarly, ALDH2 activity was decreased by 20.2% in the presence of PUGNAc in comparison with basal condition when treated under H/R, whereas its activity was increased by 16.6% in the presence of DON (*P*<0.05, Figure 4D). HG+PUGNAc decreased ALDH2 activity by 18.8% in comparison with HG condition when treated under H/R, whereas ALDH2 activity was enhanced in HG+DON group by 20.0% in comparison with HG condition (*P*<0.05, respectively. Figure 4D).

These results firmly confirmed the causal effect of ALDH2 *O*-GlcNAc modification on its activity in normal and hyperglycemia conditions under H/R settings or not.

### ALDH2 *O*-GlcNAc modification promoted 4-HNE, aldehydes, protein carbonyl accumulation and apoptosis

**Figure 5:**
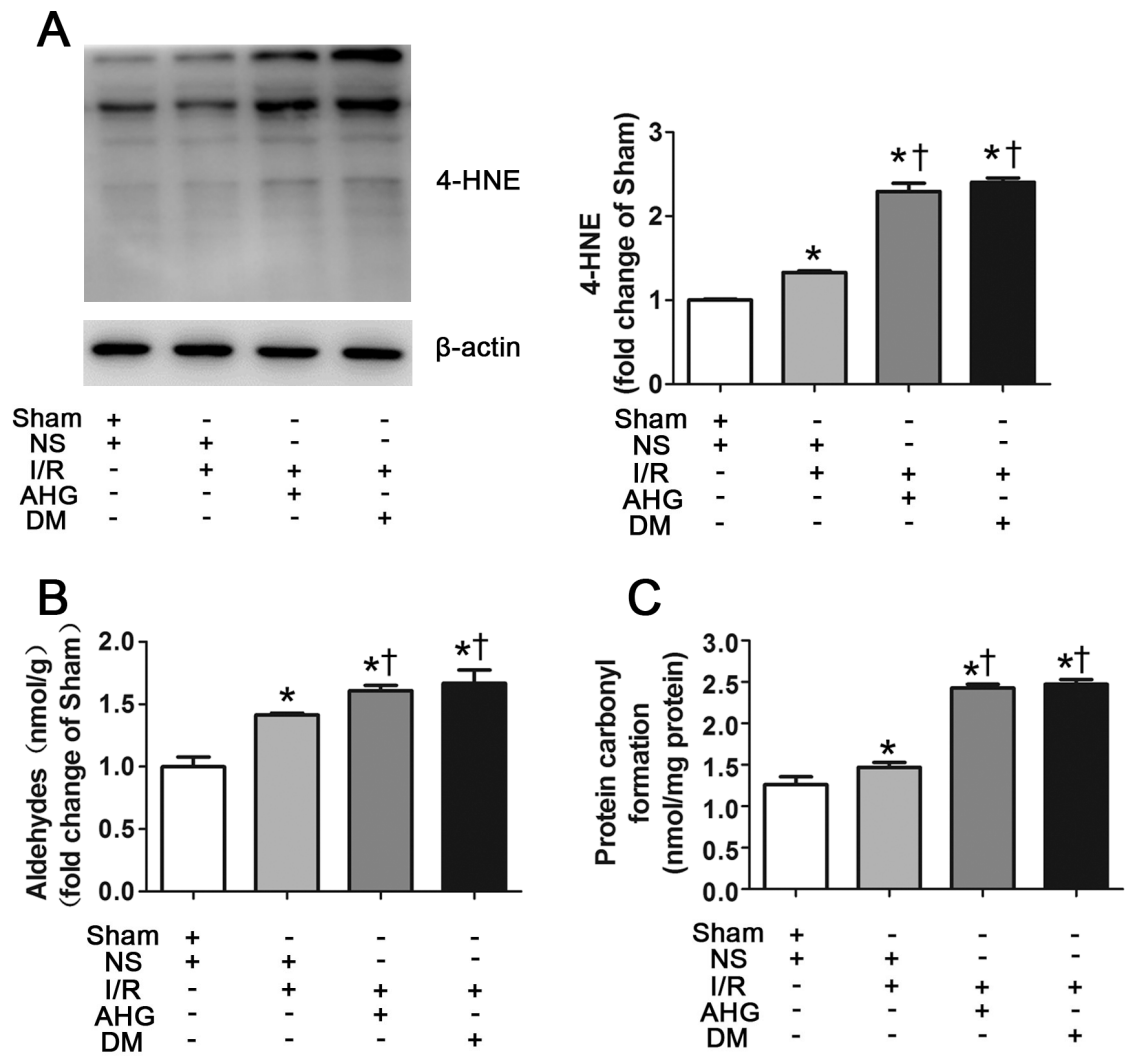
Hyperglycemia exacerbated the levels of myocardial 4-hydroxy-2-nonenal (4-HNE)-protein adducts, aldehydes and carbonyl formation after ischemia/reperfusion in rats **A**. The level of 4-HNE-protein adducts in myocardial tissue. **B**. The level of aldehydes. **C**. The level of carbonyl formation. In each graph, the mean value of Sham was expressed as 1 or 100%. Sham, sham-operated; NS, normal saline; I/R, myocardial ischemia/reperfusion; AHG, acute hyperglycemia; DM, diabetes. Data are means ± SEM of 4 to 5 samples in each group. *P<0.05 vs Sham group; †*P*<0.05 vs NS+I/R group.

**Figure 6:**
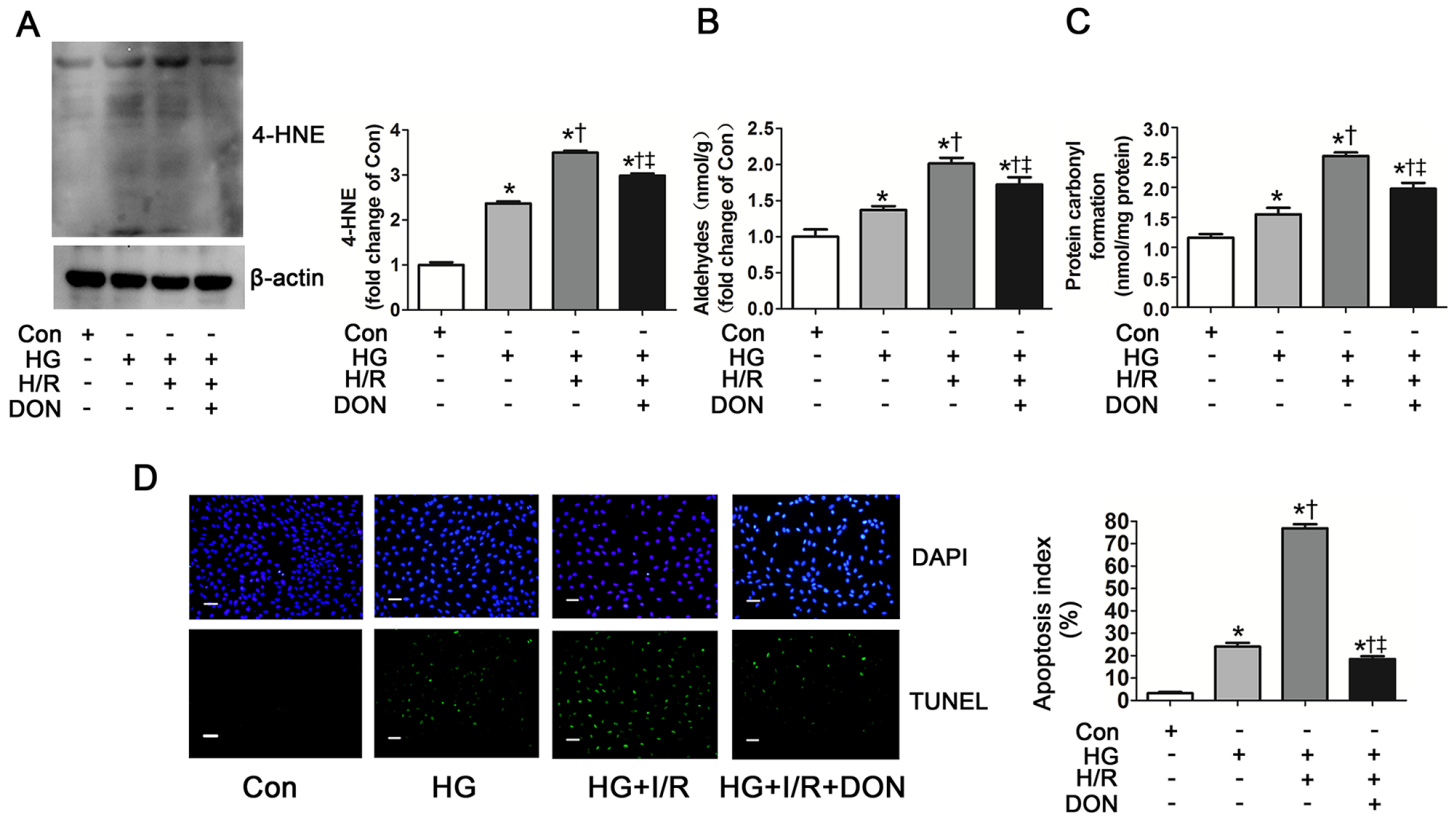
*O*-GlcNAc modification of ALDH2 contributed to 4-HNE accumulation, aldehydes and carbonyl formation and apoptosis **A**. The level of 4-HNE-protein adducts in Con, HG, HG+H/R, HG+H/R+DON groups *in vitro*. Cell lysates were separated by SDS-PAGE, and western blot was performed using specific anti-4-HNE antibody. **B**. The level of aldehydes. **C**. The level of protein carbonyl formation. **D**. Left: representative photographs of in situ detection of apoptotic cells by TUNEL staining; Right: percentage of TUNEL-positive nuclei in H9c2 cells. Scale bar, 200μm. Mannitol was used to keep the osmolarity consistent among different glucose concentrations *in vitro*. Con: normal glucose; HG: high glucose; H/R: hypoxia/reoxygenation; DON: a specific *O*-GlcNAc modification inhibitor. Data are means ± SEM of 4 to 5 samples in each group. *P<0.05 vs Con group, †*P*<0.05 vs HG group, ‡*P*<0.05 vs HG+H/R group.

The myocardial levels of 4-HNE, aldehydes and protein carbonyl formation were further evaluated as they were regulated by ALDH2 activity. The levels of 4-HNE were slightly increased in NS+I/R rat group compared with Sham group and were significantly increased in AHG+I/R group and DM+I/R group (Figure 5A). *In vitro* studies showed that 4-HNE was detectable in control group, whose levels were remarkably increased under HG conditions and they further increased in the HG+H/R group. However, 4-HNE was prominently decreased in the HG+H/R+DON group, compared with HG and HG+H/R groups, respectively (Figure 6A). Similar trends were observed for aldehydes and protein carbonyl formation. The results were shown in Figure 5 and Figure 6.

4-HNE accumulation could induce apoptosis [25, 26]. Whether ALDH2 *O*-GlcNAc modification exacerbated cell apoptosis was unclear. We also evaluated apoptosis index in this study. Compared with control group, apoptosis index was significantly increased in HG and HG+H/R groups (*P*<0.05, respectively. Figure 6D). Apoptosis index was significantly decreased in HG+H/R+DON group compared with HG and HG+H/R groups, respectively, but it was slightly higher than that in control group (Figure 6D).

These data indicate that elevated ALDH2 *O*-GlcNAc modification could promote 4-HNE, aldehydes, protein carbonyl accumulation and cellular apoptosis.

### Alda-1 removed ALDH2 *O*-GlcNAc modification and improved myocardial I/R injury

**Figure 7:**
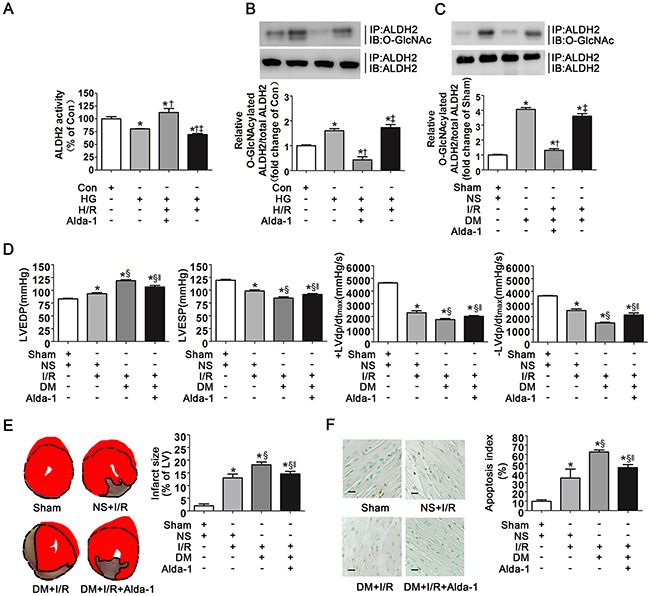
Alda-1 decreased ALDH2 *O*-GlcNAc modification and improved myocardial ischemia/reperfusion injury exacerbated by hyperglycemia **A**. ALDH2 dehydrogenase activity in Con, HG, HG+H/R, HG+H/R+Alda-1 groups *in vitro*. ALDH2 activity was determined by measuring the conversion of NAD^+^ to NADH. **B**. ALDH2 *O*-GlcNAcylation in Con, HG, HG+H/R, HG+H/R+Alda-1 groups *in vitro*. Cell lysates were immunoprecipitated with specific anti-ALDH2 antibody and then subjected to western blot using anti-*O*-GlcNAc (CDT110.6) antibody. **C**. ALDH2 *O*-GlcNAcylation in Sham, DM, DM+I/R, DM+I/R+Alda-1 groups in rats. **D**. LVEDP, LVESP, +LVdp/dt_max_ and -LVdp/dt_max_ in Sham, NS+I/R, DM+I/R, DM+I/R+Alda-1 groups in rats. LVEDP: left ventricular end diastolic pressure; LVESP: left ventricular end systolic pressure; +LVdp/dtmax, left ventricular maximal rate of pressure increase; -LVdp/dt_max_, left ventricular maximal rate of pressure decrease. **E**. Left: representative photographs of heart sections stained by 2, 3, 5-triphenyltetrazolium (TTC) in Sham, NS+I/R, DM+I/R, DM+I/R+Alda-1 groups in rats; Right: myocardial infarct size expressed as percentage of the total left ventricular area. **F**. Left: representative photographs of in situ detection of apoptotic myocytes by TUNEL staining in Sham, NS+I/R, DM+I/R, DM+I/R+Alda-1 groups in rats; Right: percentage of TUNEL-positive nuclei in heart tissue sections. Scale bar, 50μm. Mannitol was used to keep the osmolarity consistent among different glucose concentrations *in vitro*. In A-C graphs, the mean value of Con or Sham was expressed as 1 or 100%. Con: normal glucose; HG: high glucose; H/R: hypoxia/reoxygenation; Alda-1: a specific ALDH2 activator; Sham, sham-operated; DM, diabetes; I/R, myocardial ischemia/reperfusion. Data are means ± SEM of 4 to 8 samples in each group. *P<0.05 vs Con or Sham group, †*P*<0.05 vs HG or DM group, ‡*P*<0.05 vs HG+H/R+Alda-1 or DM+I/R+Alda-1 group, §*P*<0.05 vs NS+I/R group, ‖*P*<0.05 vs DM+I/R group.

Alda-1 was a specific activator of ALDH2, however, whether Alda-1 improved the activity of *O*-GlcNAcylated ALDH2 needed to be confirmed. We examined ALDH2 activity in Con, HG, HG+H/R and HG+H/R+Alda-1 conditions. Consistent with our previous observations, HG and HG+H/R significantly decreased ALDH2 activity in comparison with control group (*P*<0.05, respectively. Figure 7A). However, Alda-1 significantly improved ALDH2 activity compared with HG and HG+H/R groups, respectively.

In addition, we evaluated ALDH2 *O*-GlcNAc modification in the above groups. It was interesting to note that *O*-GlcNAcylated ALDH2 levels were significantly decreased in HG+H/R+Alda-1 group compared with HG and HG+H/R groups, respectively (*P*<0.05, Figure 7B), suggesting Alda-1 prominently inhibited ALDH2 *O*-GlcNAc modification. We observed the similar results in animal studies, that was *O*-GlcNAcylated ALDH2 was significantly decreased in DM+I/R+Alda-1group, compared with DM and DM+I/R groups, respectively (*P*<0.05, Figure 7C).

We next examined the effect of Alda-1 on cardiac function, infarct size and cardiac apoptosis. Alda-1 significantly decreased LVEDP and improved LVESP, +LVdp/dt_max_ and -LVdp/dt_max_ compared with DM+I/R group (*P*<0.05, respectively. Figure 7D). Infarct size and cardiac apoptosis were also decreased in DM+I/R+Alda-1 group (*P*<0.05, respectively. Figure 7E, 7F).

## DISCUSSION

**Figure 8:**
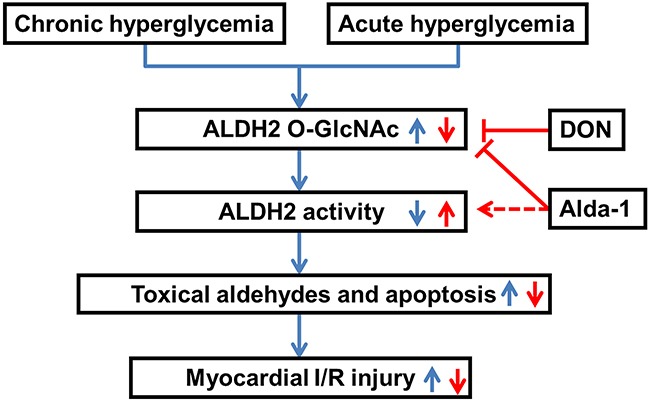
Working model of a novel mechanism for hyperglycemia-exacerbated myocardial ischemia/reperfusion injury Both diabetes-induced chronic hyperglycemia and acute hyperglycemia increased ALDH2 *O*-GlcNAc modification which decreased its activity, toxical aldehydes accumulation and cell apoptosis, thus contributing to the exacerbation of myocardial ischemia/reperfusion injury. Alda-1 reversed myocardial ischemia/reperfusion injury exacerbated by hyperglycemia through decreasing ALDH2 *O*-GlcNAc modification, as well as increasing its activity.

In the current study, we first demonstrated that ALDH2 *O*-GlcNAc modification was increased under hyperglycemia with or without myocardial I/R settings. We also made novel observations that ALDH2 *O*-GlcNAc modification inversely regulated ALDH2 activity, promoted 4-HNE accumulation, aldehydes, protein carbonyl formation and apoptosis, thus contributing to myocardial I/R injury. The study also provided further insight that Alda-1 could increase ALDH2 activity potentially through inhibiting ALDH2 *O*-GlcNAc modification. Based on these data, we proposed a working model (Figure 8) predicting that ALDH2 *O*-GlcNAc modification was a key mechanism in hyperglycemia-exacerbated myocardial I/R injury and Alda-1 exhibited a potential therapeutic target for the treatment.

The idea of this study roots in the common clinic problem that patients with acute myocardial infarction and hyperglycemia on admission have poor outcomes and high mortality. Hyperglycemia is known to increase oxidative stress, exaggerate inflammation, decrease endothelial nitric oxide production and markedly attenuate signal transduction pathways critical to endogenous cardioprotective responses [3–6]. While these mechanisms underlying hyperglycemic-exacerbation of myocardial I/R injury were reported in previous studies, other mechanisms may exist. In addition to the complicated characteristics in biological mechanisms, the interaction between hyperglycemia and myocardial I/R injury further increases the complex to investigate the mechanisms. Indeed, we found myocardial ischemia per se caused hyperglycemia through hepatic insulin signaling blunting [27], besides that hyperglycemia resulted in exacerbation of myocardial I/R injury [2, 3, 28], which formed a vicious cycle leading to poor outcomes.

In this study, we found a novel mechanism why hyperglycemia aggravated myocardial I/R injury, that is the fact that ALDH2, an important cardioprotective enzyme located in the mitochondrial matrix, was determined as a target for *O*-GlcNAc modification. The notion that mitochondrial proteins might be *O*-GlcNAc modified was first raised by the identification of a specific mitochondrial isoform of *O*-GlcNAc transferase, which promoted the formation of *O*-GlcNAc modified proteins using sugar nucleotide donors as substrates. Several mitochondrial proteins has been identified as targets for *O*-GlcNAc modification, such as NDUFA9 and COX I [14]. Our study proposed ALDH2 as another targeted protein for *O*-GlcNAc modification, which disturbed its function through inhibition of the activity. ALDH2 is a homotetramer and its activity could be regulated in various pathologies. Importantly, diabetes resulted in ALDH2 oxidation and decreased its activity whereas ischemia preconditioning by ethanol or PKC agonist induced ALDH2 phosphorylation and increased its activity [17, 18]. As a key cardioprotective enzyme which is abundant in the heart, the post-translational regulation of ALDH2 should be a big research field needing further investigation in order to clarify these regulation mechanisms and develop new therapeutic strategies.

Furthermore, we demonstrated that both acute and chronic hyperglycemia had the same effect on ALDH2 *O*-GlcNAc modification and I/R injury in myocardium. Protein *O*-GlcNAcylation is emerging as a metabolic sensor and regulator which connects cell metabolism and function. There is widely accepted notion that chronically elevated cellular *O*-GlcNAc levels is a contributing factor to the etiology of insulin resistance as well as a key mediator of glucose toxicity associated with the adverse effects of diabetes on numerous tissues including myocardium [9, 28]. However, a number of studies suggested that acute increased *O*-GlcNAcylation of proteins contribute to the tolerance of cells to a wide range of stress stimuli, such as I/R injury [29, 30], which was different from our results. It was of note that high blood glucose was induced rapidly through extra glucose administration and the levels of blood glucose exceeded diabetes-induced hyperglycemia in the setting of acute hyperglycemia. We also confirmed that ALDH2 *O*-GlcNAc modification was dose-dependent. Thus, we presumed that the extent of hyperglycemia might be critical to the *O*-GlcNAc modification of proteins and the resultant function. Indeed, we did detect a slight increase of ALDH2 *O*-GlcNAc modification in myocardial I/R procedure. This weak increased *O*-GlcNAc modification might be a stress response to the injury stimuli and led to protective signaling pathways in cells. However, when extra glucose was administrated, excessive *O*-GlcNAc modification might occur which resulted in cell dysfunction and death. Given the high energetic demand of the heart and the importance of tight control of metabolism, it was likely that the dysregulation of mitochondrial *O*-GlcNAc modification might be critical to cell dysfunction. This leaves the question whether other potential mitochondrial proteins could be *O*-GlcNAc modified needs further investigation.

While it is well established that hyperglycemia leads to increased *O*-GlcNAc modification of multiple proteins, it is important to note that increased glucose levels has various effects, including increased oxidative stress. Indeed, we previously reported that diabetes-induced hyperglycemia led to generation of ROS, which could directly inhibit ALDH2 activity. Other studies also demonstrated that hyperglycemia-induced generation of mitochondrial ROS cloud lead to increased *O*-GlcNAc modification of mitochondrial proteins [31]. Thus, although we determined the causal effect of ALDH2 *O*-GlcNAc modification on its activity, we could not rule out the possibility that ROS play a role in the process between protein post-translational modification and activity modulation, which might participated in the regulation of structure and/or function of proteins.

It was interesting to observe that Alda-1 had an effect on removal of *O*-GlcNAc from ALDH2, which had been identified as an ALDH2 activator, suggesting that Alda-1 may mediate its action on ALDH2 through decreasing *O*-GlcNAc modification as well as binding to the enzyme directly. As is mentioned above, ALDH2 is a homotetramer and each subunit has three main domains: the coenzyme-binding domain, catalytic domain and oligomerization domain [32]. The site to which Alda-1 binds is within the substrate entrance tunnel of ALDH2, which locates to the surface of this enzyme, rather than the inner catalytic site [33]. Because *O*-GlcNAc was a modification on the surface of protein, occurring after translation, we reasoned that Alda-1 should antagonize the position in ALDH2 enzyme structure where serine/threonine residues could be *O*-GlcNAcylated, thus contributing to removal of *O*-GlcNAc from ALDH2. However, the detailed mechanisms need further investigation.

Our study also demonstrated that Alda-1 improved cardiac function and decreased infarct size and cardiac apoptosis, suggesting that it played a central role in mediating cytoprotection from myocardial I/R injury under hyperglycemia. As cardiovascular risk factors such as diabetes became more popular in both developed and developing countries, Alda-1 may offer a mean of exhibiting this endogenous myocardial protection under such complex conditions.

In summary, we demonstrated that hyperglycemia increased ALDH2 *O*-GlcNAc modification, which contributed to the decrease of its activity, toxical aldehydes accumulation and cell apoptosis, thus resulting in the exacerbation of myocardial I/R injury. Alda-1 has therapeutic potential for treating the hyperglycemic exacerbation of myocardial I/R injury.

## MATERIALS AND METHODS

### Animals and experimental procedures

Adult male Wistar rats weighed 200-250g were purchased from the Department of Experimental Animals of Shandong University (Jinan, China). All animal procedures were in accordance with the National Institutes of Health Guidelines and were approved by the Animal Use and Care Committee of Shandong University.

Rats were anesthetized intraperitoneally (i.p.) with pentobarbital sodium (30mg/kg) and ventilated via a tracheostomy on a Harvard rodent respirator. A left thoracotomy was performed in the fourth and fifth intercostal space, and a reversible coronary artery snare was placed around the left anterior descending (LAD) coronary artery. Myocardial ischemia/reperfusion (I/R) was performed by tightening the snare for 30min and then loosening it for 90min.

Rats were randomly divided into four groups. Sham group: sham rats (LAD was not ligatured despite anesthetization and left thoracotomy were performed) were received saline by intravenous infusion at a rate of 4ml/kg/h throughout the experimental period. NS+I/R group: rats were treated with saline by intravenous infusion at a rate of 4ml/kg/h throughout I/R procedures. AHG+I/R group: rats were treated with glucose by a bolus intravenous injection (1.5mg/kg) and then maintained with intravenous infusion at a rate of 4ml/kg/h throughout I/R procedures. DM+I/R: diabetic rats were treated with I/R procedures. Diabetic rats were given a single injection (i.p.) of 60mg/kg streptozotocin (S0130, Sigma-Aldrich, USA) and studied 4 weeks later. For Alda-1 (SML0462, Sigma-Aldrich, USA) treatment, rats were administered with intravenous injection of Alda-1 (8mg/kg) 10min before ligation.

Serum glucose was measured by glucose analyzer every 10min during the experimental procedures. At the end of reperfusion, a micro catheter was inserted into left ventricle through right carotid artery to measure left ventricular end diastolic pressure (LVEDP) and decrease in left ventricular end systolic pressure (LVESP) and left ventricular maximal rate of pressure increase (+LVdp/dt_max_) and left ventricular maximal rate of pressure decrease (−LVdp/dt_max_). Then, the cardiac ventricles were excised rapidly, snap-frozen in liquid nitrogen and stored at -80°C until analysis. Myocardial infarct area was determined by staining with 1% 2, 3, 5- triphenyltetrazolium chloride (TTC, 17779, Sigma-Aldrich, USA) and infarct size was expressed as percentage of left ventricular area.

### Cell culture and hypoxia-reoxygenation (H/R) procedures

Rat cardiomyoblasts cell line (H9c2 SV40) was cultured in Dulbecco's Modified Eagle Medium (DMEM, 11966025, Gibco, USA ) with 1% of penicillin/streptomycin and 10% of Fetal Bovine Serum (FBS, 10082147, Gibco, USA), followed by either normal glucose (5.0mmol/L plus 25.0mmol/L mannitol) or high glucose (30.0mmol/L) conditions. In the glucose dose-dependent experiments, the glucose concentrations ranged from 5.0mmol/L to 35.0mmol/L.

The H/R procedures were performed as described previously. Briefly, H9c2 cells were exposed to 1% O_2_, 94% N_2_ and 5% CO_2_ for 12h using a modular incubator and then reoxygenation (95% air, 5% CO_2_, 37°C) lasted for 2h. H9c2 cells were exposed to 100μmol/L PUGNAc (A7229, Sigma-Aldrich, USA) to increase *O*-GlcNAc modification, or 50μmol/L DON (D2141, Sigma-Aldrich, USA) to decrease *O*-GlcNAc modification. For separate experiments, Alda-1 (10μmol/L) was given to H9c2 cells.

### Human heart specimens collection

Heart tissues were obtained from left ventricles of body donors included three patients with diabetes and three controls without diabetes. Inclusion in tissue-based studies was not restricted on the basis of age, sex, race or ethnic status. Diabetes was confirmed by medical history. The causes of death in those subjects were not heart disease. The informed contents were obtained from their relatives before body donation.

### Histological examination

Left ventricular tissues which were below ligature point were harvested. After treated by 4% buffered paraformaldehyde and embedded in paraffin, heart tissues were cut into sections of 4μm in thickness and stained with hematoxylin&eosin (H&E). Five sections from each block were selected randomly and examined with bright field microscopy. Four different areas of each section were evaluated.

### Terminal deoxynucleotide nick-end labeling (TUNEL) assay

Myocardium apoptosis was assessed with an in situ apoptosis detection kit (S7101, Millipore, USA) through the immunohistochemical method according to the manufacturer's instructions. The percentage of apoptotic cells was determined by calculating the ratio of positive-staining nuclei to the total number of nuclei.

Cardiomyocyte apoptosis was assessed with another apoptosis detection kit (11684795910, Roche, USA) using an immunofluorescence method. After TUNEL staining and DAPI staining, cells were imaged by fluorescent microscopy using 488nm excitation and 530nm emission. Cells exhibiting green fluorescence were defined as TUNEL positive, apoptotic cells. A ratio of the apoptotic cell number to the total cardiac myocytes number was calculated as the apoptotic index in each experimental group.

### Immunoprecipitation and western blot analysis

Myocardial tissues and H9c2 cells were homogenized in 50mmol/L Tris-Cl (pH=7.5), 150mmol/L NaCl, 1mmol/L EDTA, 1% NP-40 with protease inhibitors and then lysed by vortexing. Equal amounts of lysate, about 500μg protein, were incubated overnight with 2-10μg of primary antibody (ALDH2, sc-48837, 1:100, Santa Cruz Biotechnology, USA; complex IV subunit I (COX I), sc-48143, 1:100, Santa Cruz Biotechnology, USA; NDUFA9, ab14713, 1:100, Abcam, USA) rotating at 4°C, followed by incubation with 20μl of protein A/G PLUS agarose (sc-2003, Santa Cruz Biotechnology, USA) for 2h at 4°C. Immunoprecipitates were extensively washed, resuspended in 1x sample buffer, boiled for 5min and analyzed by western blot analysis.

30μg of protein was separated by 10% SDS-PAGE gel and transferred to nitrocellulose membranes, and blots were probed with antibodies against β-actin (4970, 1:1000, Cell Signaling Technology, USA), *O*-GlcNAc (CTD110.6) (9875s, 1:1000, Cell Signaling Technology, USA), 4-HNE (ab46545, 1:1000, Abcam, USA). Membranes were washed 3 times and then incubated with 1:10,000 dilution of horseradish peroxidase-conjugated secondary antibodies for 2h, and detected by using an ECL detection method. The relative intensity of the bands was quantified using the Image J software.

### Protein carbonyl formation determination

Proteins from myocardial tissues and H9c2 cells were precipitated by adding an equal volume of 20% trichloroacetic acid and centrifuged for 1min. The samples were resuspended in 10mmol/L 2, 4-dinitrophenylhydrazine solution for 30min at room temperature before 20% trichloroacetic acid was added and samples were centrifuged for 3min. The precipitate was resuspended in 6mol/L guanidine solution. The maximum value of absorbance (360 to 390nm) was read against blanks, and the carbonyl content was calculated according to the formula: absorption at 360nm ×45.45nmol/protein content (mg).

### Aldehydes determination

Aldehydes were measured using Amplite™ Fluorimetric Aldehyde Quantitation kit (10052, AAT Bioquest, Sunnyvale, CA). Myocardial tissue and H9c2 cells were homogenized in PBS, and centrifuged at 3000rpm for 10min. 50μl of the supernatant or 0-1000μM aldehyde (standards) was mixed with 50μl of AldeLight™ Blue reaction mixture. Following incubation in solid black 96-well plate for 15min at room temperature, protected from light, 25μl of reaction buffer was added, and fluorescence was detected at Ex/Em = 365/435nm.

### ALDH2 enzymatic activity measurement

The mitochondria were isolated from myocardial tissue and H9c2 cells using the Tissue Mitochondria Isolation Kit (C3606, Beyotime, Nanjing, China) and the Cell Mitochondria Isolation Kit (C3601, Beyotime, Nanjing, China). The mitochondria were sonicated, centrifuged at 11,000×g for 10 min at 4°C and the supernatant were used to measure ALDH2 activity.

Enzymatic activity of ALDH2 was determined by measuring the conversion of NAD^+^ (HY-B0445, MCE, USA) to NADH. The assays were carried out in 50mmol/L sodium pyrophosphate buffer (pH=9.5) in the presence of 10mmol/L propionaldehyde at 25°C. Propionaldehyde was oxidized in propionic acid, while NAD^+^ was reduced to NADH to measure ALDH2 activity. Production of NADH was determined by spectrophotometric absorbance at 340nm. ALDH2 activity was expressed as nmol NADH/min per mg protein.

### Statistical analysis

Data are expressed as means ± SEM. An unpaired Student's *t* test was used between two groups. A one-way analysis of variance (ANOVA) followed by Bonferroni post hoc test was used for statistical significance of multiple treatments as appropriate. A *P*<0.05 was considered statistically significant. Statistical analysis was performed with GraphPad Prism 5 (GraphPad Software, San Diego, CA, USA).

## SUPPLEMENTARY FIGURES


