# Supplementary material for: Inhibition of ALDH2 by O-GlcNAcylation contributes to the hyperglycemic exacerbation of myocardial ischemia/reperfusion injury
Source: Oncotarget. 2016 Dec 27;8(12):19413–26. doi: 10.18632/oncotarget.14297 (PMC5386694; doi:10.18632/oncotarget.14297)
Supplement: Supplementary file 1 [file oncotarget-08-19413-s001.pdf]

Inhibition of ALDH2 by *O*-GlcNAcylation contributes to the hyperglycemic exacerbation of myocardial ischemia/reperfusion injury

SUPPLEMENTARY FIGURES

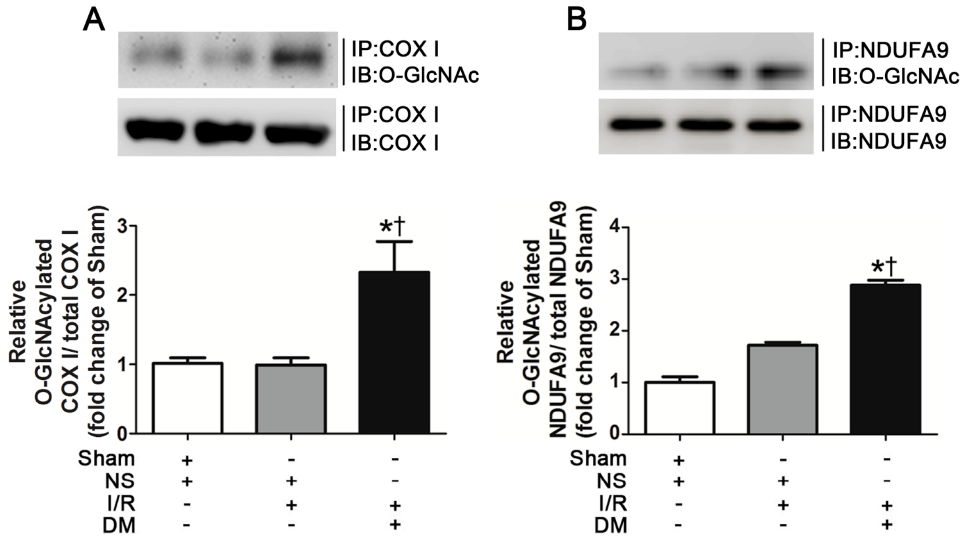

Supplementary Figure 1: Hyperglycemia increased *O*-GlcNAc modification of myocardial COX I and NDUFA9 after myocardial ischemia/reperfusion in rats. A. COX I *O*-GlcNAcylation in myocardial tissue. Tissue lysates were immunoprecipitated with specific anti-COX I antibody and then subjected to western blot using anti-*O*-GlcNAc (CDT110.6) antibody. **B.** NDUFA9 *O*-GlcNAcylation in myocardial tissue. In each graph, the mean value of Sham was expressed as 1. IP, immunoprecipitation; IB, immunoblotting; Sham, sham-operated; NS, normal saline; I/R, myocardial ischemia/reperfusion; DM, diabetes. Data are means ± SEM of 4 to 5 samples in each group. **P*<0.05 vs Sham group; †*P*<0.05 vs NS+I/R group.

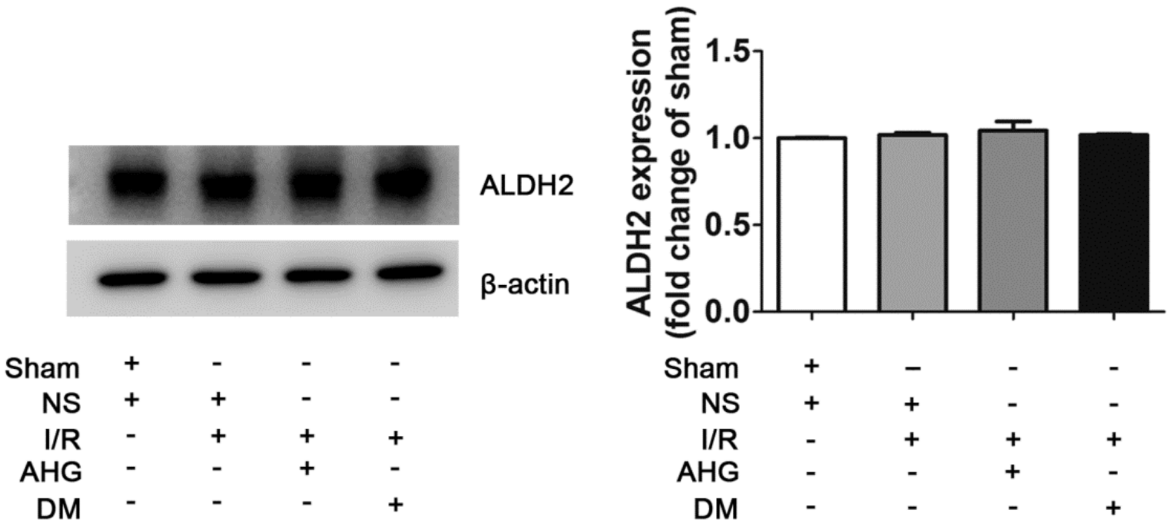

Supplementary Figure 2: ALDH2 expression in heart tissue. Tissue lysates were separated by SDS-PAGE, and western blot was performed using specific anti-ALDH2 antibody. In each graph, the mean value of Sham was expressed as 1. Sham, sham-operated; NS, normal saline; I/R, myocardial ischemia/reperfusion; AHG, acute hyperglycemia; DM, diabetes. Data are means ± SEM of 4 to 5 samples in each group. *P<0.05 vs Sham group; †P<0.05 vs NS+I/R group.

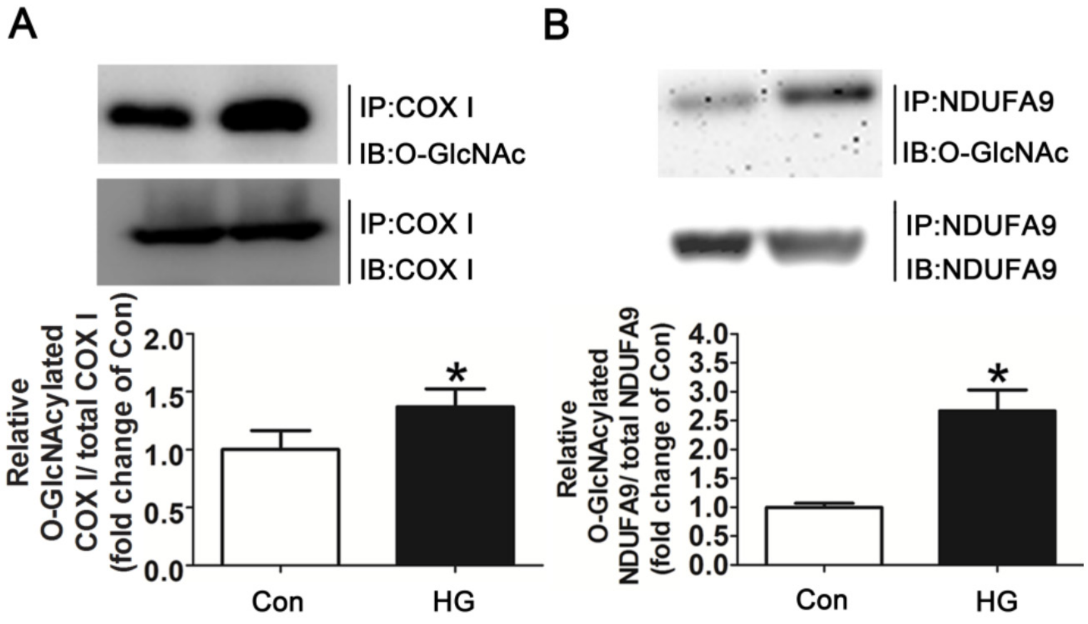

Supplementary Figure 3: Hyperglycemia increased *O*-GlcNAc modification of COX I and NDUFA9 *in vitro*. A. COX I *O*-GlcNAcylation. H9c2 cells lysates were immunoprecipitated with specific anti-COX I antibody and then subjected to western blot using anti-*O*-GlcNAc (CDT110.6) antibody. **B.** NDUFA9 *O*-GlcNAcylation. Mannitol was used to keep the osmolarity consistent among different glucose concentrations *in vitro*. In each graph, the mean value of Con was expressed as 1. IP, immunoprecipitation; IB, immunoblotting; Con: normal glucose; HG: high glucose. Data are means ± SEM of 4 to 5 samples in each group. *P<0.05 vs Con group.
